# Determinants of proper disposal of single-use masks: knowledge, perception, behavior, and intervention measures

**DOI:** 10.7717/peerj.15104

**Published:** 2023-04-06

**Authors:** Dacinia Crina Petrescu, Hamid Rastegari, Ioan Valentin Petrescu-Mag, Ruxandra Malina Petrescu-Mag

**Affiliations:** 1 Department of Hospitality Services, Faculty of Business, Babes-Bolyai University, Cluj-Napoca, Cluj, Romania; 2 Department of Economy and Rural Development, Faculty of Gembloux Agro-Bio Tech, University of Liège, Gembloux, Belgium; 3 Department of Rural Development Management, Faculty of Agriculture, Yasouj University, Yasouj, Iran; 4 Department of Engineering and Environmental Protection, Faculty of Agriculture, University of Agricultural Sciences and Veterinary Medicine Cluj-Napoca, Cluj-Napoca, Cluj, Romania; 5 Department of Environmental Science, Faculty of Environmental Science and Engineering, Babes-Bolyai University of Cluj-Napoca, Cluj-Napoca, Romania; 6 Doctoral School “International Relations and Security Studies”, Babes-Bolyai University, Cluj-Napoca, Romania

**Keywords:** Waste, COVID-19 pandemic, Pro-environmental behavior, Disposal, Single-use mask

## Abstract

**Background:**

Although many studies testify to consumer behavior’s role in the context of waste-related sustainability objectives, little research examined what people know, think, and feel about the environmental impacts of their personal protective equipment (PPE) or their behavior towards them, in general. Therefore, the present article complements existing information about the public perceptions, knowledge, and behavior of single-use masks in a context where the pandemic has put increasing pressure on waste management public services. From February to June 2020, municipal solid waste increased ten times in Romania. The study identified the factors that predicted the proper disposal of single-use masks and the measures preferred to prevent or minimize the negative impact of single-use mask waste.

**Method:**

Data from a representative sample of 705 Romanians were collected using a structured questionnaire. The data were analyzed with SPSS and SmartPLS. The Cochran’s Q test was run to determine the existence of differences between percentages of people who preferred various measures. Dunn’s test with a Bonferroni correction was used to identify the exact pair of groups where the differences were located. The study utilized structural equation models (SEM) based on at least partial squares with SmartPLS software (3.2.8) to investigate causal links between constructs. The model considered that the dependent variable (environmentally friendly behavior: proper disposal of single-use masks) could be influenced by the knowledge, perception, behavior, and demographics variables.

**Results:**

The findings indicated that knowledge of the type of material of single-use masks had a direct positive (*β* = 0.173) and significant effect on their proper disposal. The perception of mask waste impact has a negative and significant (*β* = −0.153, *p* < 0.001) impact on the proper disposal of single-use masks. This path coefficient illustrates that the worse the perceived impact of single-use masks on waste management activity, the higher the proper disposal of single-use masks. Gender has a positive (*β* = 0.115) and significant (*p* < 0.001) effect on the proper disposal of single-use masks.

**Conclusions:**

It was concluded that the 5Rs waste management approach should be reconsidered for single-use mask waste. For example, “Reuse” and the classic “Recycle” have limited applications since they may lead to virus transmission and possible infection. “Reducing” the use of single-use masks could have repercussions on one’s health. Summing up, the study outlined recommendations for effective interventions for the proper disposal of single-use masks from the perspective of behavioral studies.

## Introduction

Evidence from 1918–1919 pandemic influenza (Brienen et al., 2010), 2003 SARS epidemic, 2009 H1N1 pandemic (Mniszewski et al., 2014), and COVID-19 pandemic (Bo et al., 2021) suggests that single-use masks can be an effective nonpharmaceutical intervention to reduce the spread of viruses and thus decrease hospitalization and death rates (Eikenberry et al., 2020; Li et al., 2021). While the use of single-use masks was, already before COVID-19 pandemic, part of airborne disease prevention and control measures in East and South East Asia (Worby & Chang, 2020), in many states of the European Union (EU), it was the healthcare necessity caused by COVID-19 that has emphasized the importance of the use of single-use masks.

Even if the pandemic slows down in some parts of the world, other health crises (*e.g*., cholera in Tigray region, Ethiopia, the back of influenza in northern hemisphere) are reasons for concern that require public health measures. Thus, the production and consumption of personal protective equipment (PPE) continue. Single use masks are usually made of polypropylene (PP) (Chellamani, Veerasubramanian & Balaji, 2013), which is resistant to biological degradation and can remain in the natural environment for up to 450 years (Nghiem, Iqbal & Zdarta, 2021). The European Environment Agency (2021) estimates that about 0.75 face masks per person per day (representing 170,000 tons of face masks) were imported into the EU during this period, which is more than double than before the pandemic. More than 7,200 tons of medical waste are estimated to be generated every day by the COVID-19 pandemic, much of which are face masks (Trafton, 2021). In a recent study dedicated to the Persian Gulf, Mohamadi et al. (2023) documented that due to PPE abundance and chemical characterization, improper disposal of PPE is heavily impacting on a number of organisms. In this context, the proper disposal of single-use masks has become a real challenge for worldwide governments (Dharmaraj et al., 2021), where the proper disposal is understood as a “timely, orderly, efficient, and harmless disposal” (Zhao et al., 2021) of single-use masks.

Most single-use masks used during the pandemic are household waste and they are handled as uncontaminated municipal waste (Asim, Badiei & Sopian, 2021). However, single-use masks are potentially contaminated material and public authorities recommend specific disposal actions, such as sealing the used single-use masks into plastic bags (Torres & De-la-Torre, 2021). Sangkham (2020) who reviewed the recommendations for the disposal of sanitary facial masks in several Asian countries, reported that in Wuhan (China), the masks used were collected in special trash cans or if these were not available, they were placed in plastic bags before disposal; in South Korea, the sanitary facial masks used in non-medical units were disposed of in garbage bags labelled “Waste for incineration”. Unfortunately, many of these protective materials are not properly disposed, but rather find their way into water and terrestrial environments (Aragaw, 2020; Selvaranjan et al., 2021), raising significant issues regarding micro and nano plastic disintegration.

Although many studies testify to consumer behavior’s role in the context of waste-related sustainability objectives, little research (Botetzagias & Malesios, 2021; Liu, Duong & Nguyen, 2021) examined what people know, think and feel about the environmental impacts of their PPE or their behavior towards them, in general. Plank (2011) considers that lack of information is the biggest deterrent in forming a pro-environmental behavior. Steg & De Groot (2010) believe that an increase in people’s awareness of environmental problems caused by their behavior will make them more likely to assume responsibility for their behavior and consider that they can contribute significantly to the reduction of these environmental problems. However, it may happen that even if they are aware of what behavior produces certain results, they may sometimes not behave environmentally friendly (Donmez-Turan & Kiliclar, 2021). Public knowledge, perceptions, and behavior about single-use masks remain underexplored, and consequently, addressing this gap, the research objectives of this study were:

1) To identify factors that predict the proper disposal of single-use masks. To this end, we analyzed the public’s knowledge, perceptions, and behavior regarding single-use masks.
2) To identify people’s preferred measures to prevent or minimize the negative impact of single-use mask waste. Knowledge regarding these preferences supports effective interventions for proper disposal of single-use masks.

## Methodology

### Ethics statement

All work reported in this article was approved by the Scientific Council of Babeș-Bolyai University of Cluj-Napoca, Romania, under the Ethics Approval no. 11.426/08.09.2022.

### Data availability

The raw data from participants are downloadable as Supplemental information.

### Study area

**Figure 1:**
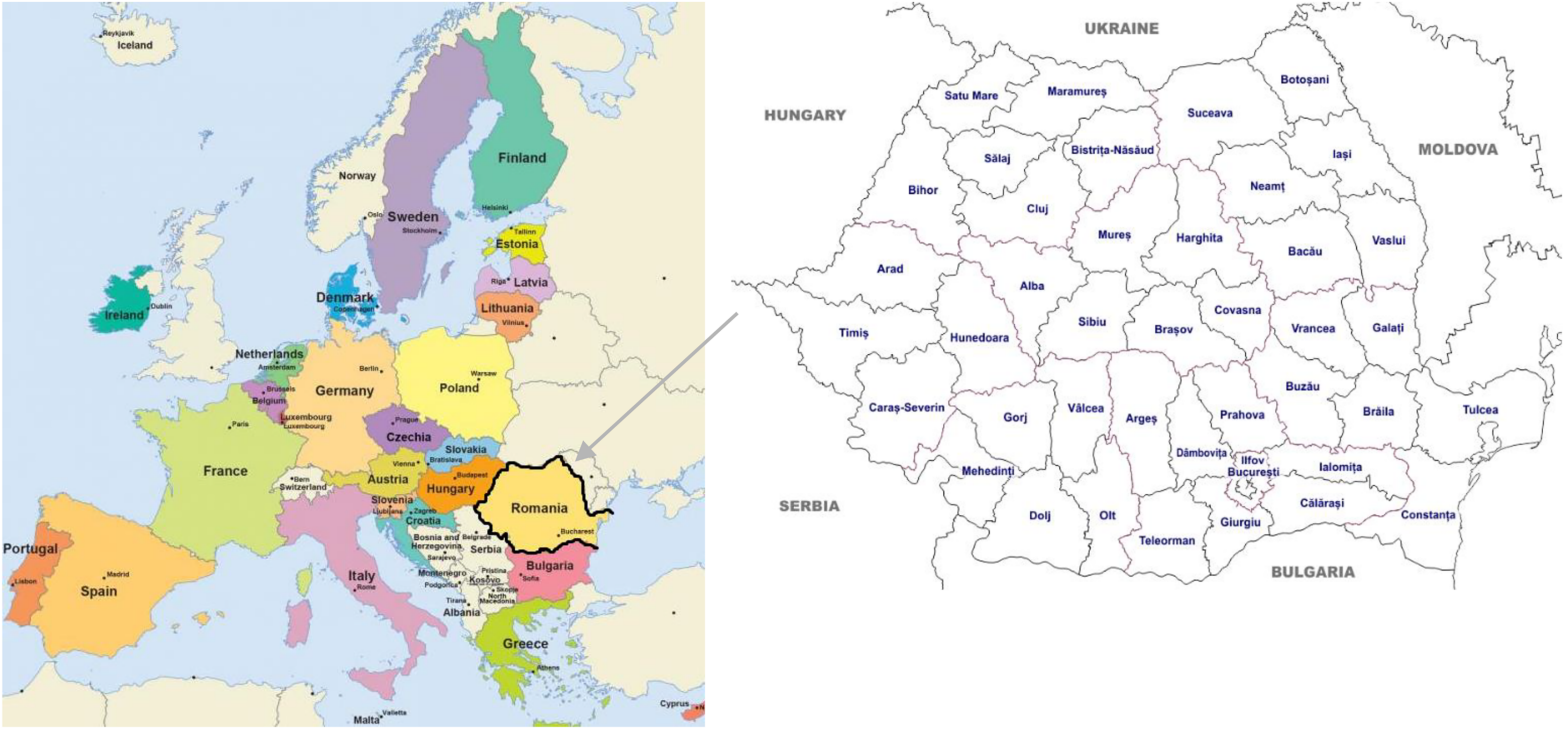
The study area and the localization of Romania in the EU. Map credits: 2023. Romania, https://d-maps.com/carte.php?num_car=5811&lang=en; the European Union, https://european-union.europa.eu/easy-read_en).

Romania is located in the southeastern part of Central Europe and, since 2007, it is member of the EU (Fig. 1). It has a population of 18,995,962 citizens, with a density of 84 people/km^2^; the urban population represents 54.6%; the median age is 43.2 years and the total land area is 230,170 km^2^ (Worldometer, 2022). Waste management is one of the biggest economic and environmental challenges for Romania. Romania has a very low municipal waste recycling rate (14%, including 7% material recycling and 7% composting) and very high landfill rate.

Worldwide, the share in municipal solid waste and the generation of municipal solid waste increased under COVID-19 conditions (Silva et al., 2021). For example, from February to June 2020, municipal solid waste increased ten times in Romania (Mihai, 2020). In 2020, the European Commission sent a letter asking Romania to close, seal, and ecologically restore the rest of the 48 illegal landfills (initially, there were 68) and to comply with the Court of Justice judgment of 2018.

### Participants and study design

The study is based on a survey that used a sample of 705 valid questionnaires and a structured questionnaire. A specialized company collected the data and written consent was obtained. Participants were members of an online national wide panel for consumer survey. The sample was representative at the country level by gender, age, and geographical distribution (considering the nine development regions of Romania) (Table A.1, Appendix). Thus, 53% of the sample were women, and the average age was 41.7 years.

The questionnaire was pre-tested twice on samples of 50 and 32 persons and adjusted. The variables investigated in this study were selected to reveal the factors that predict the proper disposal of single-use masks and public preferences for the measures to improve the proper disposal of these masks. The blue single-use mask was selected for investigation because it was the most used and disposed of one by the investigated people in the pre-test (Note 4 to Table A.2 and Table A.3, Appendix). We used a three-ply surgical face mask. This is a type of disposable mask, and it is also called a medical or surgical mask (Rubio-Romero et al., 2020).

The questionnaire was based on the study by Deng et al. (2020) that explored public’s perceptions and attitudes towards plastic and microplastics. To answer the research objectives, and similar to the Deng et al. (2020) model, the questionnaire was structured into five sections: knowledge, perceptions, behavior regarding single-use masks, preferences for certain measures to improve the proper disposal of single-use masks, and demographics (Table A.2. Appendix).

Knowledge was investigated through two questions. One aimed to determine if the respondents knew the composition material of different medical products largely used during the COVID-19 pandemic. The other one revealed their knowledge about the natural decomposition time of a single-use mask.

The section dedicated to perception included a question related to people’s perceptions of the impact of disposed single-use masks on waste management activity.

The investigation of the behavior was done through four questions about the frequency of use (number of masks used/month) of single-use masks, the reasons to use them instead of other masks (health *vs*. other), the way to use them (single-use *vs*. reuse), and the proper disposal of single-use masks (percentage of masks disposed of in improper/illegal places). We included the reasons for wearing single-use masks in the behavior section because it supported the understanding of the behavior and was in line with the Deng et al. (2020) model. In the Deng et al. (2020) model, the dependent variable was people’s willingness to reduce microplastic emissions.

In the present case, reducing single-use masks may not be a desirable action for health-safety reasons, both for the users and for the people around them. Instead, in this study, the proper disposal of single-use masks is the behavior that has environmental benefits. Consequently, the dependent variable was the proper disposal of single-use masks. This was asked in the form of the reversed question to “Proper disposal” (Table A.2, Appendix) to reduce the social desirability bias, according to which people tend to select the option that reflects a socially desirable behavior (Crowne & Marlowe, 1960).

Respondents were also asked to express their preferences for five measures to prevent or minimize the negative impact of single-use mask waste. The tested measures were: (a) information and education campaigns to raise public awareness of the large amount of medical waste that is generated and the danger to nature and human health; (b) more restrictive legislation to impose harsher penalties for those who unproperly dump this waste; (c) development of facilities (certain types of containers/garbage bins) for the safe storage of this waste, which are within reach of citizens (inside and when leaving institutions, shops, *etc*.); (d) use of biodegradable materials; and (e) recycling. The last section was dedicated to demographic variables: gender, age, living environment, education, and monthly income (more details are included in Table A.2, Appendix, Table A2).

The data were analyzed with SPSS and SmartPLS. The Cochran’s Q test was run to determine the existence of differences between percentages of people who preferred various measures. Dunn’s test with a Bonferroni correction was used to identify the exact pair of groups where the differences were located.

### Model description

This study utilized structural equation models (SEM) based on at least partial squares with SmartPLS software (3.2.8) to investigate causal links between constructs. When the investigation model comprises constructs with properties such as a single indicator formative and reflective, Hair et al. (2016) and Garson (2016) recommend using PLS-SEM. The model considered that the dependent variable “(Environmentally friendly behavior: proper disposal of single-use masks)” could be influenced by the following variables: knowledge, perception, behavior, demographics (listed in Table A.2, Appendix).

## Results

### Knowledge, perception, behavior, and preferred measures

The level of knowledge about the medical product’s type of material was average for wet wipes and masks and high for the rest of the products (Table A.2, Figure A.1A, Appendix). The decomposition time for the single-use mask was underestimated on average at the sample level (120 years) (Figure A.1B, Appendix), and only around 3% of people gave an estimation close to the 450 years decomposition time indicated by the studies (Nghiem, Iqbal & Zdarta, 2021).

The negative impact of masks waste on waste management activity was perceived as slightly high (Figure A.3, Appendix). Regarding the behavior, respondents used 26 single-use masks/month on average (Figure A.4, Appendix), and 36.2% of the people reused them (Figure A.6, Appendix). More than half of the respondents used them mainly for health reasons (Figure A.5, Appendix). Most of discarded single-use masks were disposed of correctly (Figure A.7, Appendix). The measures preferred by most people to prevent or minimize the negative impact of single-use mask waste were “Information and education campaigns”, “More restrictive legislation”, and “Development of facilities” (Table A.2, Figure A.8, Appendix).

Cochran’s Q test (Cochran, 1950) was run to determine if the percentage of people who preferred one measure differed from the percentage of people who preferred another (Laerd Statistics, 2017). A statistically significantly difference was found, χ2(4) = 483.319, *p* < 0.0005. Pairwise comparisons were performed using Dunn (1964) procedure with a Bonferroni correction for multiple comparisons. Adjusted *p*-values are presented. The results showed that 50.6% of people preferred the measure “Use of biodegradable materials”. This percentage was statistically lower than the percentage of people who preferred the first three options and higher than the ones that preferred “Recycling”. In addition, the percentage of people who preferred the “Recycling” option was lower than all the rest. All these differences were statistically significant with *p* = 0.000. No statistically significant difference exists between the percentages of people who preferred the first three measures.

### Measurement model

The standardized root mean square residual (SRMR) is a measure of model fit in PLS, and it was below 0.05, indicating the fit of the model. In this study, reflective constructs for evaluation hypotheses are used. Tables 1 and 2 provide the criteria used for reflective constructs, composite reliability (CR), average variance extracted (AVE), and heterotrait-monotrait (HTMT).

**Table 1:** Measurement properties of reflective constructs.

Constructs	AVE	CR
1) Age	1	1
2) Behavior 1: Frequency of use	1	1
3) Behavior 2: Reasons to use	1	1
4) Behavior 3: Waste reduction behavior	1	1
5) Behavior 4: Proper disposal	1	1
6) Education	1	1
7) Gender	1	1
8) Income	1	1
9) Knowledge 1: Material	1	1
10) Knowledge 2: Years decomposition	1	1
11) Living environment	1	1
12) Perception: Waste impact	1	1

**Note:**

AVE, average variance extracted; CR, composite reliability (CR)

**Table 2:** Heterotrait-monotrait (HTMT) distribution of reflective constructs.

Constructs	1	2	3	4	5	6	7	8	9		10	11	12
1) Age													
2) Behavior 1: Frequency of use	0.004												
3) Behavior 2: Reasons to use	0.108	0.044											
4) Behavior 3: Waste reduction behavior	0.025	0.317	0.088										
5) Behavior 4: Proper disposal	0.045	0.047	0.049	0.017									
6) Education	0.179	0.038	0.188	0.007	0.113								
7) Gender	0.053	0.061	0.121	0.081	0.137	0.051							
8) Income	0.06	0.07	0.111	0.029	0.041	0.376	0.054						
9) Knowledge 1: Material	0.151	0.069	0.005	0.013	0.198	0.072	0.132	0.042					
10) Knowledge 2: Years decomposition	0.086	0.005	0.007	0.05	0.036	0.039	0.064	0.004	0.014				
11) Living environment	0.108	0.004	0.085	0.112	0.038	0.22	0.031	0.204	0.001		0.018		
12) Perception: Waste impact	0.159	0.049	0.002	0.017	0.191	0.066	0.035	0.04	0.104		0.045	0.056	

### Structural model

**Figure 2:**
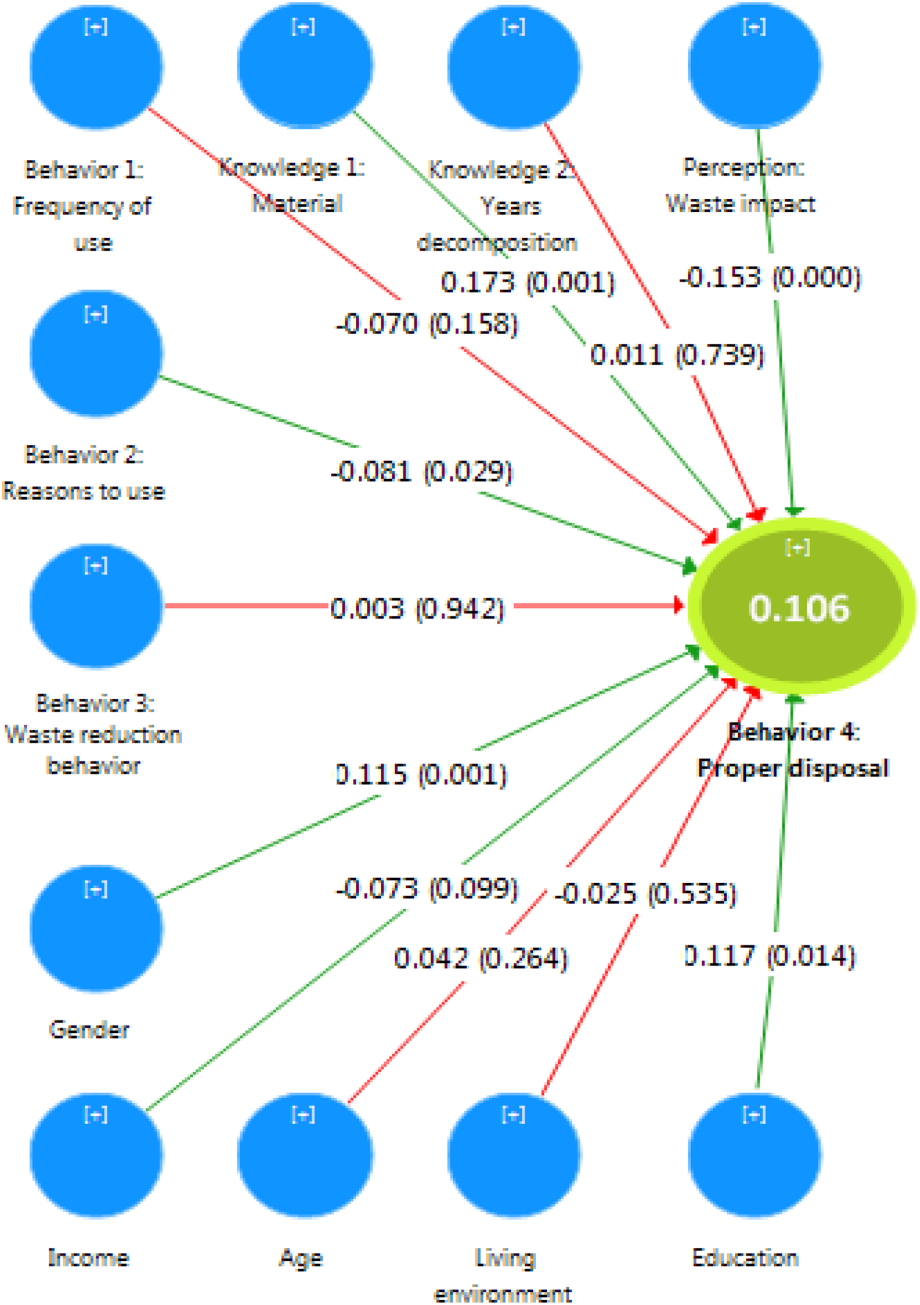
Assessing PLS-SEM structural model of proper disposal of single-use masks.

Figure 2 illustrates the results of the structural model estimate, such as total effects and *p*-values. The significance of the structural paths is validated by resampling 500 and 5,000 times using the bootstrap method. Fig. 2 shows the significant paths that can be identified between the independent variables and the dependent variable.

The results show that “Knowledge 1: Material” has a direct positive (*β* = 0.173) and significant (*p* < 0.001) effect on the proper disposal of single-use masks (Fig. 2). This positive coefficient path indicates that an increase in the knowledge of the material of PPE is associated with an increase in the proper disposal of single-use masks. Moreover, this construct has the highest path coefficient among all other constructs of the model research. This means that knowledge of the material is the most important construct among the independent factors.

The perception of mask waste impact has a negative and significant (*β* = −0.153, *p* < 0.001) impact on the proper disposal of single-use masks. This path coefficient illustrates that the worse the perceived impact of single-use masks on the waste management activity, the higher the proper disposal of single-use masks. The variable “Reasons” to wear single-use masks has a positive and significant (*β* = −0.081, *p* < 0.05) effect on the proper disposal of single-use masks. This coefficient path shows that when people wear these masks for health-related reasons, their proper disposal of single-use masks increases.

Gender has a positive (*β* = 0.115) and significant (*p* < 0.001) effect on the proper disposal of single-use masks. This means that between men and women, women are more likely to properly dispose of single-use masks. Education has a positive (*β* = 0.117) and significant (*p* < 0.05) impact on the proper disposal of single-use masks. Therefore, when the level of education increases, the proper disposal of single-use masks also increases. Income has a negative (*β* = −0.073) and significant (*p* < 0.1) effect on the proper disposal of single-use masks, showing that an increase in the income level is associated with a lower proper disposal of single-use masks.

According to Table 3, some constructs do not have a significant path coefficient with the proper disposal of single-use masks, and these are “Knowledge 2: Years decomposition”, “Behavior 1: Frequency of use”, “Behavior 3: Waste reduction behavior”, age, and living environment. Therefore, we can conclude that these constructs do not have a statistically significant effect on the proper disposal of single-use masks.

**Table 3:** Structural estimates.

Path (from → to)	Path Coef.	T-values	*p*-values
Age → Behavior 4: Proper disposal	0.042^NS^	1.117	0.264
Behavior 1: Frequency of use → Behavior 4: Proper disposal	−0.07^NS^	1.411	0.158
Behavior 2: Reasons to use → Behavior 4: Proper disposal	−0.081**	2.18	0.029
Behavior 3: Waste reduction behavior → Behavior 4: Proper disposal	0.003^NS^	0.073	0.942
Education → Behavior 4: Proper disposal	0.117**	2.46	0.014
Gender → Behavior 4: Proper disposal	0.115***	3.196	0.001
Income → Behavior 4: Proper disposal	−0.073*	1.649	0.099
Knowledge 1: Material → Behavior 4: Proper disposal	0.173***	3.323	0.001
Knowledge 2: Years decomposition → Behavior 4: Proper disposal	0.011^NS^	0.333	0.739
Living environment → Behavior 4: Proper disposal	−0.025^NS^	0.621	0.535
Perception: Waste impact → Behavior 4: Proper disposal	−0.153***	4.469	0.001

**Note:**

***p < 0.001; **p < 0.05; *p < 0.1; NS: not significant.

Regarding the structural model, the PLS-SEM method was applied to analyze Stone–Geisser’s Q^2^, R^2^, and path coefficients to observe the predictive accuracy and power of the model, as well as the strength of the relationship between constructs in the determined paths (Table 4). The obtained values indicate an acceptable predictive power of the model.

**Table 4:** Q^2^, R^2^, and R^2^_Adjusted_ of the research model.

	SSO	SSE	Q^2^ (=1-SSE/SSO)	R square	Adjusted R square
Proper disposal	705	649	0.078	0.106	0.092

## Discussion

The recent increase in the use of single-use masks triggered by the COVID-19 pandemic intensified the existing concerns about plastic and microplastic pollution and the need for effective solid waste management strategies at all levels (Das et al., 2021; Nghiem, Iqbal & Zdarta, 2021), from citizen level to country and international levels. In response, this study analyzed the public’s knowledge, perceptions, and behavior regarding single-use masks to identify the factors that predict the proper disposal of single-use masks. We also revealed people’s preferred measures to prevent or minimize the negative impact of single-use masks waste. Regarding the measurement model, the results indicate that the model fits the data well because the SRMR was below 0.05 (Hair et al., 2016; Henseler & Sarstedt, 2013). According to Table 2, the values of all mentioned indices became significant at an acceptable level. This implies that all indicators of each construct have an appropriate connection. The HTMT criteria presented in Table 2 further indicate that each indicator belongs to its construct and differs significantly from other constructs. HTMT values below 0.8 are recommended for this purpose by Hair et al. (2016). In relation to the structural model, Table 4 illustrates that the value of Q^2^ in the form of cross-validated redundancy for the model’s endogenous constructs is positive with a value of 0.078. This shows that the model has predictive accuracy (Alexander et al., 2012; Hair et al., 2016). The obtained R^2^_adj._ is 0.098, which indicates that the model has acceptable predictive power (Henseler & Sarstedt, 2013).

In the following, the relevance of the findings is discussed considering the constructs of the model. One of the model’s constructs was the “knowledge”. The findings of this study indicated that like Deng et al. (2020) results, the knowledge of composition material influenced the environmentally friendly behavior, which was the proper disposal of masks in this study. This suggests that increasing people’s knowledge about the type of materials, such as visible labelling, can lead to better masks disposal. However, less than half of investigated people knew what the correct material type of single-use masks was (Table A.2., Appendix). This is in line with (Akarsu, Madenli & Deveci, 2021) and (Asim, Badiei & Sopian, 2021) conclusions, who warned that the lack of awareness was a significant problem of mask waste pollution and that this happened because most people considered that masks were pieces of clothes harmless to the environment and not plastic products.

The other tested knowledge variable (knowledge about the natural decomposition time) did not influence the proper disposal of the masks. Furthermore, most people were too optimistic about the decomposition time, with 71.2% of them considering a lower decomposition time or not knowing it (Table A.2., Appendix; the percentage includes all who did not answer “450 years” + 50 years and “I don’t know”). Studies demonstrated the persistence of mask residues in terrestrial, aquatic, and aerial systems; furthermore, they pointed out their long-term harmful impacts because, for example, they were ingested by wild animals, and they changed soil properties and processes (Knicker & Velasco-Molina, 2022). Additionally, they are a long-term source of microplastics (Fadare & Okoffo, 2020; Shen et al., 2021).

“Perceptions” were also included in the model. The perception of single-use masks impact on waste management activity was lower than expected. Interviewed people assigned a 4.2 score to the negative impact of these masks on the waste management activity, which means a relatively high impact, but not in the range of “extremely” or “very” high one (Table A.2, Appendix). Given the growing concern signaled in the media and scientific literature about single-use mask littering in the environment, the difficulties posed on the waste management activity, and the high number of masks visible in improper places (outside the waste bins), we expected people to perceive a more serious negative impact. For example, Akarsu, Madenli & Deveci (2021) estimated (based on UNCTAD (2020) data) that 841,000 metric tons of medical waste (face masks and gloves) were generated per month during the pandemic. They also found that three cities in Türkiye (inhabited by 4 million people) produce 2.5 million used face masks daily, which is the equivalent of 10 tons per day (Akarsu, Madenli & Deveci, 2021). In comparison, the present study indicated a 40% higher amount (14 tons per day) generated by the same amount of people (four million) and this includes only the blue single-use masks. More precisely, Romanians reported an average of 26 single-use masks used per month, resulting in almost 2,000 tons of mask waste monthly at the country level or nearly 66 tons/day (considering the country’s population of around 19 million people).

“Behavior” was the model’s construct consisting of four questions. Most tested people (64.8%) use and prefer the single-use mask because they consider it protects their health better than other masks. This suggests that if a biodegradable alternative is offered, then this should look like the old PP masks in order to be easily accepted, or efficient information campaigns should persuade people that the biodegradable masks can protect their health at least as well as the PP ones. Studies (Lee et al., 2021) and day-by-day observations signal that people reuse single-use masks when they should dispose of them. In the present study, as expected, many people (36.2%) reused the single-use masks, which could negatively affect their health and the health of people close by. Even if this behavior has a positive environmental impact on waste generation by reducing it, it cannot be considered desirable. Therefore, solutions should be found to compensate for its adverse effects by stimulating people to use them correctly or by replacing them with reusable alternatives.

The fact that most of the masks used were disposed of properly is a positive aspect from health and environmental points of view. However, the social desirability bias that drives people to respond in a socially acceptable way (in this case, to declare that they properly dispose the single-use masks) imposes caution in considering that this finding accurately reflects the reality. In other countries, for example, in Bangladesh, less than half of the participants in a survey are reported to properly dispose of masks and other PPE, dumping the household wastes, including sanitary masks, near the house (Islam et al., 2021). Thus, we should also be alert about the risk of improper disposal and its associated consequences, that potentially increases the spread of coronavirus (Mol & Caldas, 2020). The new normality of wearing single-use masks and improper disposal will spill over into environmental pollution related to micro and nanoplastic and follow-up waste (Sangkham, 2020). Awareness for litter prevention, introduction of eco-friendly single-use masks, and stricter regulation of illegal dumping activities are an integrative approach to the waste problem caused by the considerable quantity of PPE suggested in the waste management literature (Heidbreder et al., 2019; Vanapalli et al., 2021).

The lack of awareness and medical waste bins were signaled in the scientific literature (Akarsu, Madenli & Deveci, 2021) as main causes of the current problem regarding pollution with single-use masks. In the present study, people preferred the first three measures (“Information-education campaigns”, “Restrictive legislation”, and “Dedicate bins”, Table A.2, Appendix) to prevent or minimize the negative impact of single-use mask waste, with no statistically significant difference between them. The match between the main causes of single-use masks pollution and people’s preference for intervention measures revealed by this study increases the chances of success in reducing pollution. Specialists proposed the development of bio-based solutions as a viable alternative for PP masks when it is accompanied by a positive result of a life cycle assessment from the environmental impact perspective (compared with other alternatives, such as article) and validation from the stakeholders, such as industry (Silva et al., 2020). For example, bio-based polymers can make masks more eco-friendly (Akarsu, Madenli & Deveci, 2021). Despite its eco-friendly nature, this measure was second among the preferences of tested people. In studies of other plastic items, such as plastic bags (usually perceived as made of plastic), the use of eco-friendly alternatives was the preferred measure by households (O’Brien & Thondhlana, 2019). For the present study, it is possible that the confusion regarding the composition of these masks generated a lower preference rate than expected. This lack of awareness, the underestimation of the decomposition time revealed by this study, as well as possible concerns regarding their contamination with pathogens may determine people to be reluctant to their recycling, which ranked the last. This result suggests that awareness of the effectiveness and safety of various recycling options should be increased. For example, studies found that the addition of single-use masks materials to asphalt mixtures is safe and improves the performance of asphalt (Goli & Sadeghi, 2022).

“Gender”, “education”, and “income” were the demographic variables that predicted the proper disposal of single-use masks. In the present study, women were more likely to properly dispose of single use masks. In the same geographical region, a research by Ganczak et al. (2021) found that the correct mask usage was gender dependent, with more Polish women using a mask in the proper manner. An explanation could come from Capraro & Barcelo (2020) explanation that men were more likely to view mask wearing as a “sign of weakness” compared to women. The statistical analysis indicated that the level of education influenced the proper disposal of single-use masks. Other studies (Botelho, 2012; Minelgaitė & Liobikienė, 2019) also illustrated that education is essential for a proper waste management behavior. For example, a higher level of education was a significant contributor to the adoption of prevention measures (*e.g*., safe disposal of masks and gloves, handwashing) to control the spread of the SARS-CoV-2 virus in Bangladesh (Islam et al., 2021). Income was often cited as an influential variable in the adoption of prevention and protective behaviors during the COVID-19 pandemic (Irigoyen-Camacho et al., 2020; Hearne & Niño, 2022). In the present case, an increase in the level of education was associated with a lower “Proper disposal” of single-use masks. This signals that the formal education is not always enough to foster a simple pro-environmental behavior such as the proper disposal of single-use masks. More should be discovered about what type of messages can be effective in the case of educated people or what are the incentive that can stimulate them to properly discard the single-use masks.

The findings revealed the decisive factors for the proper disposal of single-use masks among Romanians. On the basis of the above findings and the targeted discussion, we suggest several practical contributions from the government and managerial perspective that could encourage future sustainable waste behaviors in Romania. From a managerial perspective, a proper disposal of single-use masks is beneficial at both the individual and the business levels. The PLS-SEM analysis revealed that the variables that influenced the proper disposal of single-use masks were “Knowledge 1: Type of material of medical protection products”, “Perception of the impact of single-use mask waste”, “Reason for using single-use masks”, gender, education, and income. In the specific case of Romania, the upper-level decision makers must add positivity to the perception of residents of their contribution to fighting all types of waste production and improve perceived control abilities for a proper disposal behavior. For stakeholders interested in the sustainability of waste management, such as NGOs, media, and local authorities, this result highlights the fact that they should assign priority to actions that increase awareness of the type of masks material and negative impact of single-use mask waste (over the actions linked to variables that did not significantly influence the proper disposal of single-use masks, Fig. 2). In addition, they should also stress the efficiency of the health protection of these masks in people’s minds, as those who use them for health reasons are more likely to discard them properly. Not least, a single-use mask garbage classification could increase their proper disposal, which can reduce the environmental burden of landfilling. Hence, the entry of single-use plastic products to coastal areas of particular biodiversity and ecosystemic relevancy should be prohibited. Sustainable alternatives to the extensive use of PPE caused by the COVID-19 pandemic could mitigate the waste generation. In this sense, Rakib et al. (2021) extensively presented a wide variety of solutions that range from PPE waste converted into gas and liquid fuels through pyrolysis to the use of degradable plastics, such as biobased and biodegradable plastics (Torres et al., 2019). Still, the availability of biobased disposable masks is still very limited (Selvaranjan et al., 2021). Other research reported on the incorporation of single-use masks as additives to pavements base (Saberian et al., 2021). Thus, despite having more sustainable alternatives, often single-use products are preferred with dramatic consequences on the environment and human health. Therefore, the prohibition of their use in areas of ecosystemic relevancy could be a solution both for Romania and other countries (Hatami et al., 2022).

The finding that knowledge and education influence the proper disposal could pave the way for significant changes in the educational curriculum that should be valued as an instrument to respond to society’s expectations at gaining capabilities to address the environmental, social, health, and economic aspects of waste management. This should result in a paradigm shift in the perception of waste not as “garbage”, but as a “source of income” and in the awareness that waste is a direct consequence of human activity, with direct and positive changes in our behavior. Formal education and information campaigns that foster the 5Rs waste management culture in general (Reduce, Refuse, Reuse, Repurpose, and Recycle), or more complex “Rs” formulas such as the 10Rs (Rahman et al., 2021) can empower people to become more responsible in managing the waste. Likewise, informal education supported by skill development and learning-by-doing approaches is the premise for creating environmental awareness that is one step towards building the 5Rs culture. The zero-waste movement, grounded in waste prevention and minimalist consumption behavior, inspires many people to contribute to waste problems. However, community involvement alone is not always enough to obtain the expected outcomes due to the persistence of economic, social, and institutional constraints. Consequently, an alternative to the traditional waste management system must be the participatory management approach, where local authorities and people have co-responsibility and co-management for a better waste management system. In Romania, likewise it was suggested for other countries (Onyena et al., 2021), environment scientists, public authorities, business sector, and lay people will need to approach a structured collaborative management model to support SDGs and fight all types of MPs in the environment. In addition to proper disposal, proper use of masks is equally important. Consequently, new research should focus on revealing effective ways to determine people to use masks according to producers’ recommendations. More research should be done on women’s contribution to single-use mask waste disposal behavior, as they play an essential role in daily household waste management (Almasi et al., 2019) and overall family hygiene.

## Conclusions

The present research answers the need for more information about people’s perception, behavior, knowledge, and proper disposal related to single-use masks in a context where the COVID-19 pandemic has put increasing pressure on waste management public services. We revealed the factors that predict the proper disposal of single-use masks and the public’s preferences for the measures to improve their disposal. The findings allow us to highlight the following aspects related to the proper disposal of single-use masks.

Thus, the 5Rs waste management approach should be totally reconsidered. “Reuse” and the classical “Recycle” have limited applications since they may lead to virus transmission and possible infection (Almulhim et al., 2021). “Repurpose” (upcycle) and “Refuse” could be problematic since the use of single-use masks is related to a health emergency, and their wear is often imposed by law. Consumption of single-use masks is correlated with the dynamics of COVID-19 spread or other health and environmental emergencies (*e.g*., air pollution). Therefore, “Reducing” the use of the single-use masks could have repercussions on one’s health.

Depending on the regulations in force and existing waste management facilities, the valorization of single-use mask waste can be done in various ways (*e.g*., pyrolysis—common chemical valorization technique for plastic waste and thermomechanical valorization (Battegazzore, Cravero & Frache, 2020; Asim, Badiei & Sopian, 2021)). Consequently, we face challenges in considering the disposal of single-use masks that is closely related to their separation, storage, and collection with the overall aims of reducing plastic waste and pollution. In addition to technical advances in the field of solid waste management, behavioral studies are needed in order to better understand the proper disposal of single-use masks, as people’s behavior could be either a deterrent or stimulus to the successful implementation of the waste management system.

Finally, the research should be considered in the light of several limitations. This study used data on self-reported behavior, and although self-reported behavior is considered a good indication of the actual behavior, a difference between these two can exist. A bigger sample that is representative on more variables, in addition to gender and age used here (such as education and living environment), can increase the accuracy of the results. Psychological constraints are reported (Minn, Srisontisuk & Laohasiriwong, 2010) as deterrents of pro-environmental behavior towards proper waste management. Therefore, future studies can investigate the contribution of various psychological factors (*e.g*., self-esteem) to people’s path dependency that is visible in changing existing attitudes and behaviors toward public care for a clean environment.

## Supplemental Information

10.7717/peerj.15104/supp-1 Supplemental Information 1 Factors that predict the proper disposal of single-use masks.

10.7717/peerj.15104/supp-2 Supplemental Information 2 Raw data.

10.7717/peerj.15104/supp-3 Supplemental Information 3 Questionnaire.

10.7717/peerj.15104/supp-4 Supplemental Information 4 Appendix.

## Additional Information and Declarations

### Data Availability

The following information was supplied regarding data availability: The raw data is available in the Supplemental File.
