# Supplementary figures and images for: Determinants of proper disposal of single-use masks: knowledge, perception, behavior, and intervention measures
Source: PeerJ. 2023 Apr 6;11:e15104. doi: 10.7717/peerj.15104 (PMC10083004; doi:10.7717/peerj.15104)

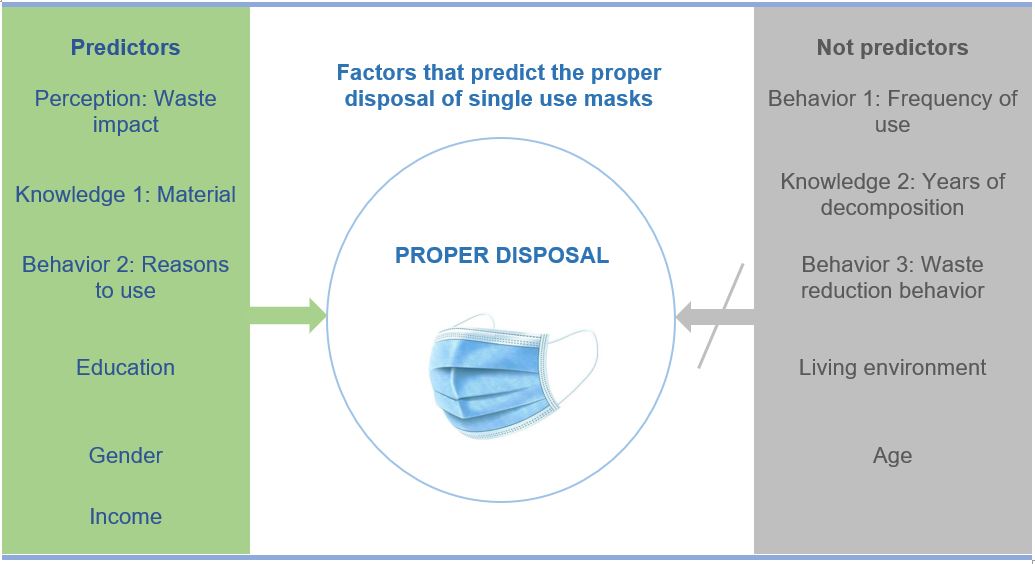

Supplement: Supplemental Information 1 [file peerj-11-15104-s001.jpg]
