# Supplementary material for: Determinants of proper disposal of single-use masks: knowledge, perception, behavior, and intervention measures
Source: PeerJ. 2023 Apr 6;11:e15104. doi: 10.7717/peerj.15104 (PMC10083004; doi:10.7717/peerj.15104)
Supplement: Supplemental Information 3 [file peerj-11-15104-s003.docx]

Questionnaire

| 1. Please indicate the type of material you think the products below are usually made of: | |
| --- | --- |
| 1.1. Antibacterial wet wipes  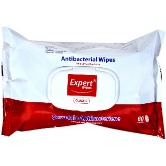 | a) It is usually made of 100% synthetic / plastic material  b) It is usually made of 100% natural material (e.g., cotton, linen, hemp, paper, leather, glass)  c) It is usually made of a mixture of synthetic and natural materials  d) I do not know |
| 1.2. Single-use mask 1  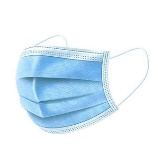 | a) It is usually made of 100% synthetic / plastic material  b) It is usually made of 100% natural material (e.g., cotton, linen, hemp, paper, leather, glass)  c) It is usually made of a mixture of synthetic and natural materials  d) I do not know |
| 1.3. Cloth mask 2  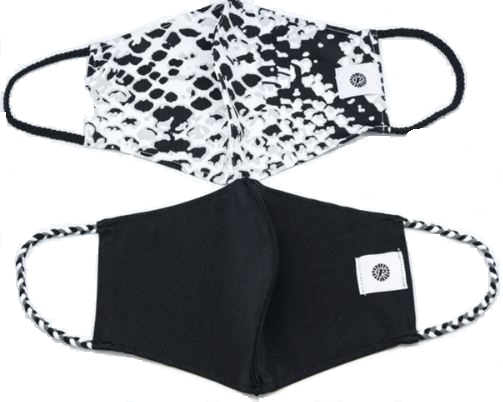 | a) It is usually made of 100% synthetic / plastic material  b) All options are usually available in various stores: 100% synthetic, 100% natural, and a mixture of both  c) It is usually made of 100% natural material (e.g., cotton, linen, hemp, paper, leather, glass)  d) It is usually made of a mixture of synthetic and natural materials |
| 1.4. Single-use mask 3  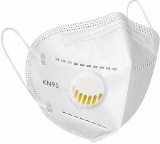 | a) It is usually made of 100% synthetic / plastic material  b) It is usually made of 100% natural material (e.g., cotton, linen, hemp, paper, leather, glass)  c) It is usually made of a mixture of synthetic and natural materials  d) I do not know |
| 1.5. Single-use mask 4  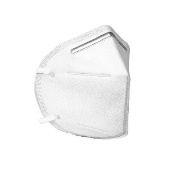 | a) It is usually made of 100% synthetic / plastic material  b) It is usually made of 100% natural material (e.g., cotton, linen, hemp, paper, leather, glass)  c) It is usually made of a mixture of synthetic and natural materials  d) I do not know |
| 1.6. Transparent face shield  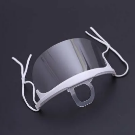 | a) It is usually made of 100% synthetic / plastic material  b) It is usually made of 100% natural material (e.g., cotton, linen, hemp, paper, leather, glass)  c) It is usually made of a mixture of synthetic and natural materials  d) I do not know |
| 1.7. Single-use gloves  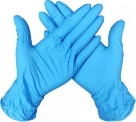 | a) It is usually made of 100% synthetic / plastic material  b) It is usually made of 100% natural material (e.g., cotton, linen, hemp, paper, leather, glass)  c) It is usually made of a mixture of synthetic and natural materials  d) I do not know |
| 1.8. Single-use protection suit  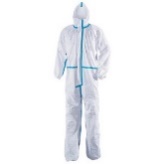 | a) It is usually made of 100% synthetic / plastic material  b) It is usually made of 100% natural material (e.g., cotton, linen, hemp, paper, leather, glass)  c) It is usually made of a mixture of synthetic and natural materials  d) I do not know |
| 1.9. Single-use shoe covers  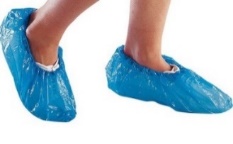 | a) It is usually made of 100% synthetic / plastic material  b) It is usually made of 100% natural material (e.g., cotton, linen, hemp, paper, leather, glass)  c) It is usually made of a mixture of synthetic and natural materials  d) I do not know |
| 1.10. Transparent shield in a car  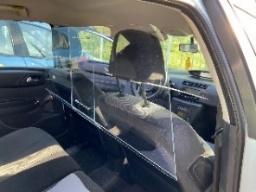 | a) It is usually made of 100% synthetic / plastic material  b) It is usually made of 100% natural material (e.g., cotton, linen, hemp, paper, leather, glass)  c) It is usually made of a mixture of synthetic and natural materials  d) I do not know |
| 1.11. Transparent shield in a classroom/ office/ front desk etc.  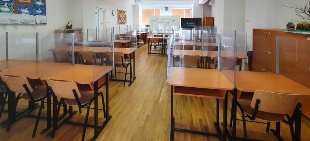 | a) It is usually made of 100% synthetic / plastic material  b) It is usually made of 100% natural material (e.g., cotton, linen, hemp, paper, leather, glass)  c) It is usually made of a mixture of synthetic and natural materials  d) I do not know |
| 2. The following questions of the questionnaire refer to the type of mask you see below ^4^.  In how many years do you think that the mask below decomposes naturally?  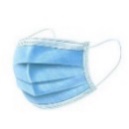 | …………………. (Open answer) |
| 3. Indicate how big do you consider the impact of the waste of single-use masks is on the waste management activities (collection, recycling, landfill) in your city. Choose a value between 0 and 10, where 0 = Catastrophic impact, ….., 5 = Average negative impact,…., 10 = No negative impact  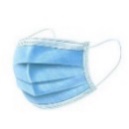 | …………………. (Open answer) |
| 4. Write down the number of single-use masks that you used in a month, on average, during the period March 2020- March 2022.  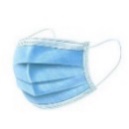 | …………………. (Open answer) |
| 5. Indicate the main reason why you use single-use masks instead of other mask types.  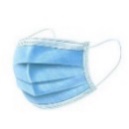 | a) Health reasons: They protect your health better than other masks (e.g., cloth ones)  b) Other reasons (e.g., They are cheaper than the cloth ones; They are easily available in stores; You got used to wearing them; It is mandatory by law; Other reason) |
| 6. What do you usually do with the single-use masks after you used them for the first time?  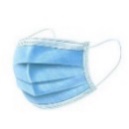 | a) Single-use (You throw them away after a single-use)  b) Reuse (e.g., You reuse them again after washing them; You reuse them after sterilizing them with ultraviolet light; You put them in a certain place and reuse them again, but without washing or disinfecting them; Other way to reuse) |
| 7. Of the single-use masks that you used during March 2020- March 2022, how many did you thrown directly onto the street or in other improper/ illegal places?  Write a percentage between 0% and 100% (for example: 0% = No masks were thrown by you on the street, in the water, etc.; 50% = You threw half of the masks that you used on the street, in the water, etc.; 100% = You threw all the masks that you used on the street, in the water, etc.)  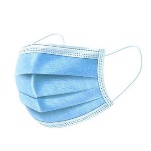 | …………………. (Open answer) |
| 8. What do you think would be the best measures to minimize or prevent the negative impact of single-use mask waste?  You can choose all answers that apply to you.  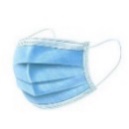 | a) Information and education campaigns to raise public awareness of the large amount of medical waste that is generated and the danger to nature and human health;  b) More restrictive legislation to impose harsher penalties for those who improperly dump this waste;  c) Development of facilities (certain types of containers / garbage bins) for the safe storage of this waste, that are within the reach of citizens (inside and when leaving institutions, shops, etc.);  d) Use of biodegradable materials;  e) Recycling. |
| 9. Gender. Please select your option | a) M;  b) F;  c) Other  d) Prefer not to say |
| 10. Age. Please mention your age in years. | …………………. (Open answer) |
| 11. Living environment. Please select your option | a) Urban;  b) Rural |
| 12. Education. Please select your option | a) 8 years of education;  b) 10 years of education;  c) 12 years of education;  d) college or higher |
| 13. Income. Please select your option | a) Max. 1000 Ron/ month (<210 Euro);  b) 1001-3000 Ron/ month (211-620 Euro);  c) 3001-5000 Ron/ month (212-1030 Euro);  d) Above 5000 Ron/ month (>1030 Euro) |
