# Supplementary material for: Determinants of proper disposal of single-use masks: knowledge, perception, behavior, and intervention measures
Source: PeerJ. 2023 Apr 6;11:e15104. doi: 10.7717/peerj.15104 (PMC10083004; doi:10.7717/peerj.15104)
Supplement: Supplemental Information 4 [file peerj-11-15104-s004.docx]

**Appendix**

**Table A.1:**

**Demographic profile of the respondents.**

| **Variable** | **Category** | | | | |
| --- | --- | --- | --- | --- | --- |
| Gender (%) | *M* | *F* | | | |
|  | 47 | 53 | | | |
|  | *Years* | | | | |
| Age (mean) | 41.68 | | | | |
| Residence (%) | *Urban* | *Rural* | | | |
|  | 80.6 | 19.4 | | | |
| Education (%) | *8 years of education* | *10 years of education* | *12 years of education* | *College and higher* | |
|  | 1.7 | 4.3 | 29.9 | 64.1 | |
| Monthly income (%) | *Maximum 1000 Ron (<210 Euro)* | *1001-3000 Ron*  *(211-620 Euro)* | *3001-5000 Ron (212-1030 Euro)* | | *Above 5000 Ron (>1030 Euro)* |
|  | 15.3 | 45.7 | 26.1 | | 12.9 |

**Table A.2:**

**Investigated variables and results.**

| **Research objectives** | **Investigated variables** (IV= independent variable; DV= dependent variable) | | **Question in the questionnaire** | **Answer options** | **Results** |
| --- | --- | --- | --- | --- | --- |
| **RO1: To identify factors that predict the proper disposal of single-use masks** | **1. Knowledge** | |  |  |  |
|  |  | 1.1. Knowledge 1: Type of material of medical protection products (IV)  (Knowledge 1: Material) ^1^ | Please indicate the type of material you think the product below is usually made of ^2^: | For masks 1, 3, 4, and the rest of products ^3^:  a) It is usually made of 100% synthetic / plastic material  b) It is usually made of 100% natural material (e.g., cotton, linen, hemp, paper, leather, glass)  c) It is usually made of a mixture of synthetic + natural materials  d) I do not know  For mask 2 ^3^:  a) It is usually made of 100% synthetic / plastic material  b) All options are usually available in various stores: 100% synthetic, 100% natural, and a mixture of both  c) It is usually made of 100% natural material (e.g., cotton, linen, hemp, paper, leather, glass)  d) It is usually made of a mixture of synthetic + natural materials | Results are listed below the table |
|  |  | 1.2. Knowledge 2: Years of decomposition for single-use masks (IV)  (Knowledge 2: Years decomposition) | The following questions of the questionnaire refer to the type of mask you see below ^4^.  In how many years do you think that the mask below decomposes naturally?  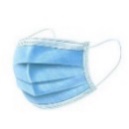 | Open answer | Average: 120 years  Grouped answers:  a) Less than 1 year: 0.8%  b) 1-10 years: 7.2%  c) 11-50 years: 20.1%  d) 51-100 years: 28.5%  e) 101-200 years: 11.8%  f) 200-1000 years: 22.6%  g) over 1000 years: 6.2%  h) I do not know: 2.8% |
|  | **2. Perception** | |  |  |  |
|  |  | 2.1. Perception of the impact of single-use masks waste (IV)  (Perception: Waste impact) | Indicate how big do you consider the impact of the waste of single-use masks is on the waste management activities (collection, recycling, landfill) in your city.  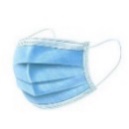 | Open answer  …………….  Choose a value between 0 and 10, where 0 = Catastrophic impact, ….., 5 = Average negative impact,…., 10 = No negative impact | Average score – Waste management: 4.2 |
|  | **3. Behavior** | |  |  |  |
|  |  | 3.1 Frequency of use of single-use masks (IV)  (Behavior 1: Frequency of use) | Write down the number of single-use masks that you used in a month, on average, during the period March 2020- March 2022.  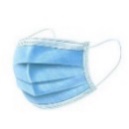 | Open answer  ……………… | Average no: 26 masks/ month |
|  |  | 3.2 Reason for using single-use masks (IV)  (Behavior 2: Reasons to use) | Indicate the main reason why you use single-use masks instead of other mask types.  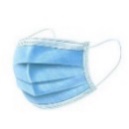 | a) Health reasons: They protect your health better than other masks (e.g., cloth ones)  b) Other reasons (e.g., They are cheaper than the cloth ones; They are easily available in stores; You got used to wearing them; It is mandatory by law; Other reason) | - Health reasons: 54.2%  - Other reasons: 45.8% |
|  |  | 3.3 Waste reduction behavior for single-use masks: Type of use (single *vs* reuse) (IV)  (Behavior 3: Waste reduction behavior) | What do you usually do with the single-use masks after you have used them for the first time?  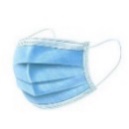 | a) Single-use (You throw them away after a single-use)  b) Reuse (e.g., You reuse them again after washing them; You reuse them after sterilizing them with ultraviolet light; You put them in a certain place and reuse them again, but without washing or disinfecting them; Other way to reuse) | - Single-use: 64.8%  - Reuse: 36.2% |
|  |  | **3.4 Environmentally friendly behavior: proper disposal of single-use masks (DV)**  (Behavior 4: Proper disposal) | Of the single-use masks that you have used during March 2020- March 2022, how many did you thrown directly onto the street or in other improper/ illegal places?  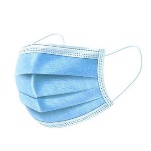 | Open answer ^5^  ……………….  Write a percentage between 0% and 100% (for example: 0% = No masks were thrown by you on the street, in the water, etc.; 50% = You threw half of the masks that you used on the street, in the water, etc.; 100% = You threw all the masks that you used on the street, in the water, etc.) | Average percentage: 94.8% (percentage calculated with reversed codes: it indicates the average percentage of masks correctly disposed) |
| **RO2: To identify people’s preferred measures for improving proper disposal of single use masks** | **4. Action options to improve the activity of waste management of single-use medical masks** | | What do you think would be the best measures to minimize or prevent the negative impact of single- use mask waste?  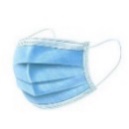 | Multiple possible answers:  a) Information and education campaigns to raise public awareness of the large amount of medical waste that is generated and it’s danger to nature and human health;  b) More restrictive legislation to impose harsher penalties for those who improperly dump this waste;  c) Development of facilities (certain types of containers / garbage bins) for the safe storage of this waste, that are within the reach of citizens (inside and when leaving institutions, shops, etc.);  d) Use of biodegradable materials;  e) Recycling. | First place: a) 61.7%; b) 62.3%; c) 63.8%; (no statistically significant differences between them)  Second place: d) 50.6%;  Third place: e) 16.9%. |
|  | **5. Demographic variables** | |  |  |  |
|  |  | 5.1. Gender (IV) | Please select your option | a) M; b) F; c) N | M: 47%; F:53%; N: 0% |
|  |  | 5.2. Age (IV) | Please select your option | Open answer | Average: 41.7 years |
|  |  | 5.3. Living environment (IV) | Please select your option | a) Urban; b) Rural | Urban: 80.6%  Rural: 19.4% |
|  |  | 5.4. Education (IV) | Please select your option | a) 8 classes; b) 10 classes; c) 12 classes; d) college or higher | 8 classes: 1.7%; 10 classes: 4.3%; 12 classes: 29.9%; college or higher: 64.1% |
|  |  | 5.5. Income (IV) | Please select your option | a) Max. 1000 Ron/ month (<210 Euro); b) 1001-3000 Ron/ month (211-620 Euro); c) 3001-5000 Ron/ month (212-1030 Euro); d) Above 5000 Ron/ month (>1030 Euro) | Max. 1000 Ron/ month (<210 Euro): 15.3%; 1001-3000 Ron/ month (211-620 Euro): 47.5%; 3001-5000 Ron/ month (212-1030 Euro): 26.1%; Above 5000 Ron/ month (>1030 Euro): 12.9%. |

| ^1^ The short names used in the PLS-SEM are mentioned between parentheses for each variable.  ^2^ Pictures 1.1.1. to 1.1.11. were presented one by one to the respondents: | | | |
| --- | --- | --- | --- |
| 1.1.1. Antibacterial wet wipes  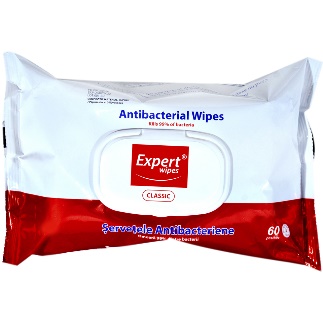 | 1.1.2. Single-use mask 1  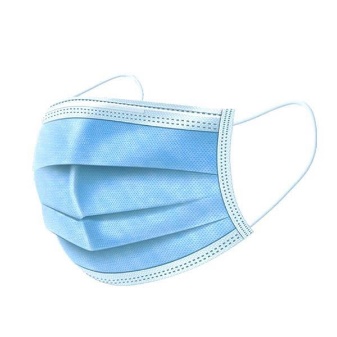 | 1.1.3. Cloth mask 2 ^6^  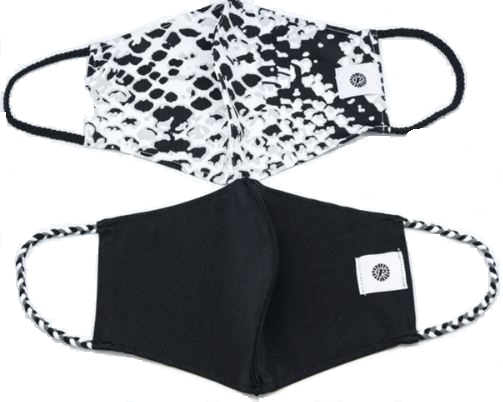 | 1.1.4. Single-use mask 3  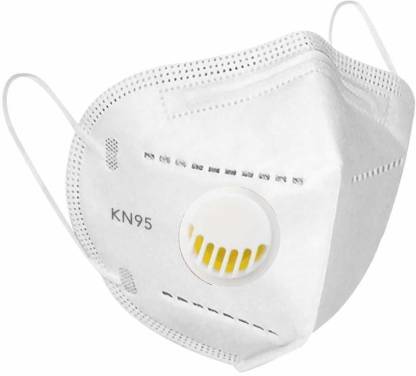 |
| a) Correct answer [“a)”]: 30.6% | a) Correct answer [“a)”]: 48.8% | a) Correct answer [“b)”]: 49.9% | a) Correct answer [“a)”]: 39.7% |
| 1.1.5. Single-use mask 4:  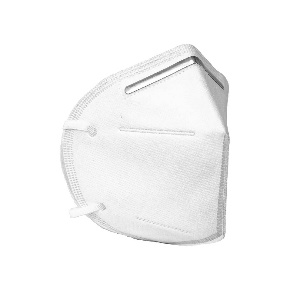 | 1.1.6. Transparent face shield (or a similar product):  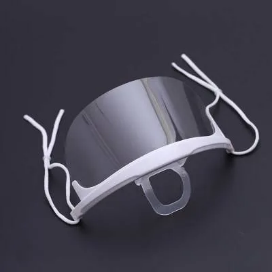 | 1.1.7. Single-use gloves:  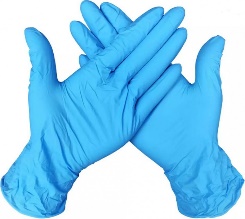 | 1.1.8. Single-use protection suit:  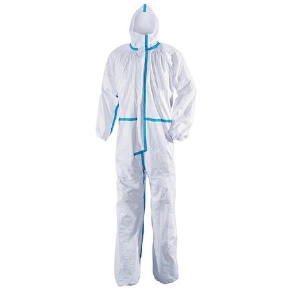 |
| a) Correct answer [“a)”]: 36.0% | a) Correct answer [“a)”]: 89.1% | a) Correct answer [“a)”]: 89.2% | a) Correct answer [“a)”]: 64.5% |
| 1.1.9. Single-use shoe covers  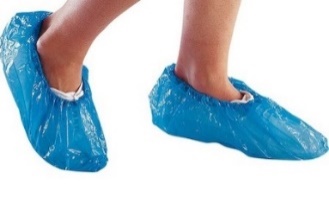 | 1.1.10. Transparent shield in a car  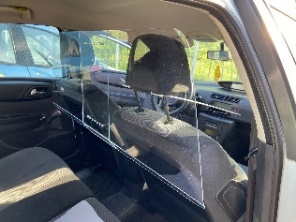 | 1.1.11. Transparent shield in a classroom/ office/ front desk etc.  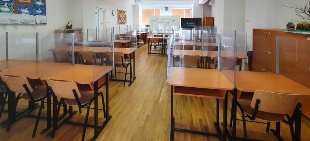 |  |
| a) Correct answer [“a)”]: 90.8% | a) Correct answer [“a)”]: 87.7% | a) Correct answer [“a)”]: 8.9% |  |
| ^3^ A score one (1) was given to a person who gave the correct answer and zero (0) to a person who gave the wrong answer or did not know.  ^4^ A pre-test was made on 112 persons regarding the number of masks they used on average per month during March 2020-March 2022 considering seven categories of masks (Table A.3., Appendix). Two questions were asked: (1) “What type of mask did you use the most frequently during March 2020-March 2022? Consider the number of days/hours when you used a certain type”; (2) “Now, consider the number of masks of the following categories. Indicate the mask type of which you used and discarded the highest number of pieces/ month”. The blue mask (mask 2, Table A.3., Appendix) was the one with the highest number of answers for both questions: this was the most frequently used one, by 73 people (65.2%), and the highest number of disposed pieces/ month belonged to this category, being mentioned by 96 people (85.7%). Consequently, this was used in the questionnaire (Table A.2., Appendix). It is worth mentioning that in Romania, the law did not impose the use of a specific mask type (e.g., KN95 or FFP2), as it happened in other EU countries.  ^5^ Calculated with reversed codes: 0 became 100, 1 became 99, etc.  ^6^ We checked a sample of 62 cloth masks for adults and kids from various shops (in Romania; online and in store; supermarkets and corner shops): 38 of them (61.3%) were labeled “100% cotton”, 11 of them (17.7%) were a mixture of cotton and synthetic, eight (12.9%) were 100% synthetic, and five (8.1%) had no label. | | | |

**Table A.3:**

**Type of masks tested ^7^** **for use frequency and number of discarded pieces per month in the pre-test**

| 1.1.1. Mask 1  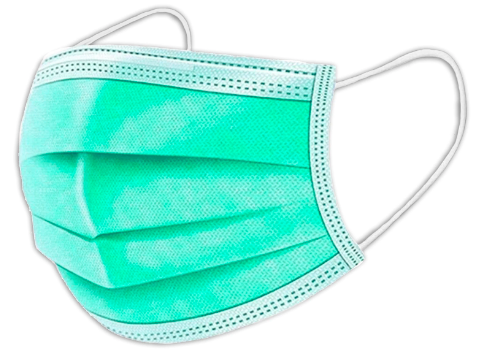 | 1.1.2. Mask 2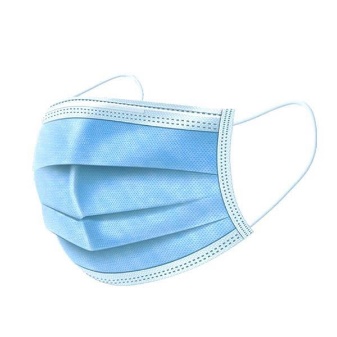 | 1.1.3. Mask 3  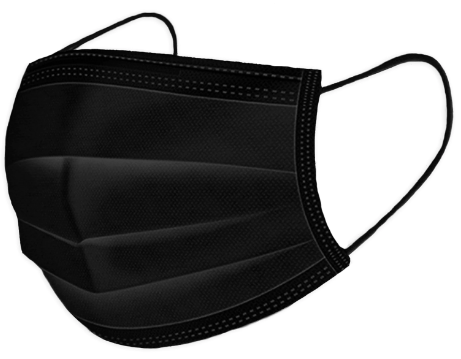 | 1.1.4. Cloth mask 4  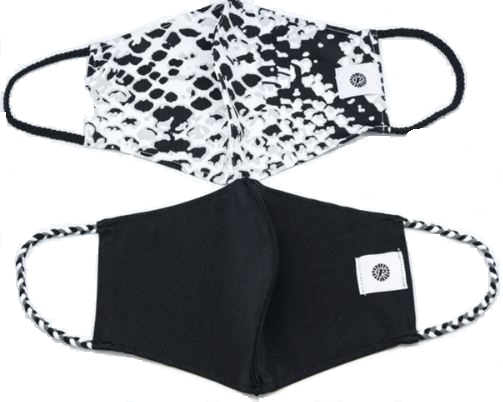 | 1.1.5. Mask 5  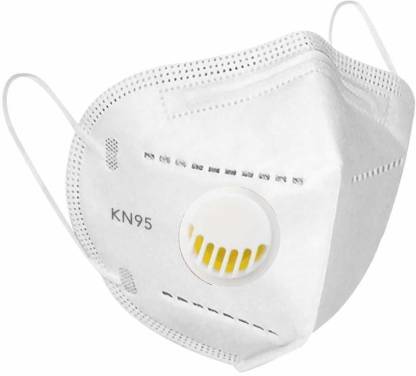 |
| --- | --- | --- | --- | --- |
| 1.1.6. Mask 6:  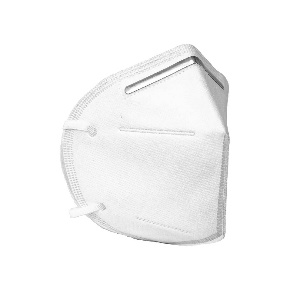 | 1.1.7. Mask 7:  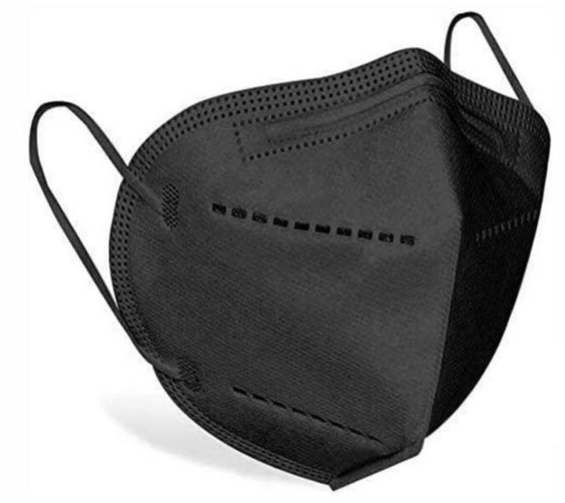 | 1.1.8. Transparent face shield (or a similar product):  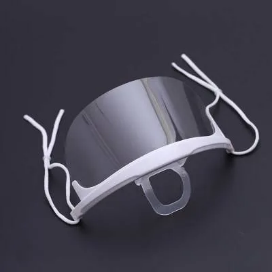 | 1.1.9. Other |  |
| ^7^ The masks included in the pre-test were selected as follows. Prior to their inclusion in the questionnaire, in three days of the week, at three moments of the day (8-10, 12-14, and 19-22), researchers noted during 15 minutes the types of masks they saw that people were wearing (450 observations). The observations took place on public and private places, and in work and non-work situations, such as: on the street, on children’s playground, in shops, in waiting rooms of private clinics, notary offices, banks, and postal offices. All mask types that counted for at least 2% of the observations were included in the pre-test. | | | | |


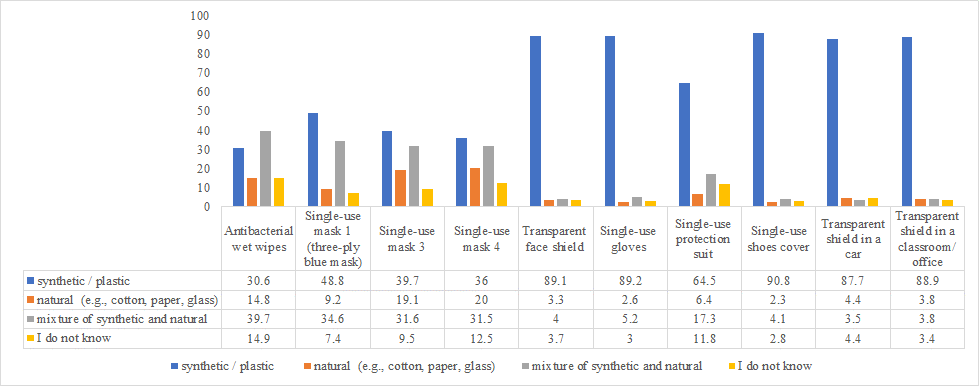


**(a)**

**(b)**

**Figure A.1: Percentage of persons who gave a certain answer for the variable “Knowledge 1: Type of material of medical protection products”.** (**a) Antibacterial wet wipes; Single-use mask 1 (three-ply blue mask); Single-use mask 3; Single-use mask 4; Transparent face shield; Single-use gloves; Single-use protection suit; Single-use shoe covers; Transparent shield in a car; Transparent shield in a classroom/ office (correct answer: “synthetic / plastic”); (b) Single-use mask 2 (cloth masks) (correct answer: “all options”).**

**Figure A.2: Percentage of persons who gave a certain answer for the variable “Knowledge 2: Years of decomposition for single-use masks (three-ply blue mask)”.**

0 = Catastrophic impact, ….., 5 = Average negative impact,…., 10 = No negative impact

**Figure A.3: Percentage of persons who perceived a certain level of impact of single-use masks waste (three-ply blue mask).**

**Figure A.4: Average number of single-use masks used in a month during the period March 2020- March 2022 (standard deviation=21.8; min=0; max=200).**

**Figure A.5: Percentage of persons who had a certain category of reasons for wearing single-use masks.**

**Figure A.6: Percentage of people who used the single-use masks one time *vs* reusing.**

**Figure A.7: Percentage of single-use masks correctly disposed of by people during the period March 2020-March 2022 out of the total number of used masks.**

**Figure A.8: Percentage of persons who considered certain policy measures as the best ones.**
